# Use of N-Acetylcysteine in Psychiatric Conditions among Children and Adolescents: A Scoping Review

**DOI:** 10.7759/cureus.1888

**Published:** 2017-11-29

**Authors:** Sadiq Naveed, Afshan Amray, Ahmed Waqas, Amna M Chaudhary, Muhammad W Azeem

**Affiliations:** 1 Psychiatry, KVC Hospitals, Kansas; 2 Department of Medicine, Dow Medical College, Dow University of Health Sciences; 3 Department of Psychiatry, CMH Lahore Medical College and Institute of Dentistry; 4 Psychiatry, Nishtar Medical College Hospital, Multan, Pakistan; 5 Department of Psychiatry, Sidra Medical and Research Center; Weill Cornell Medical College, Cornell University

**Keywords:** n-actylecysteine, pediatric, children, review, autism, addiction, tourette syndrome, trichotillomania, cannabis, pathological onychophagia

## Abstract

N-acetylcysteine (NAC) is a well-known antidote for acetaminophen toxicity and is easily available over the counter. It has antioxidant and anti-inflammatory properties and an established tolerance and safety profile. Owing to its neuroprotective effects, its clinical use has recently expanded to include the treatment of different psychiatric and non-psychiatric disorders. Although a number of randomized controlled trials have documented the clinical evidence for NAC, there are no reviews that summarize the evidence. The present scoping review summarizes the study designs, the patient characteristics, the evidence and the limitations in randomized controlled trials designed to explore the efficacy of NAC for psychiatric conditions in the pediatric population.

## Introduction and background

N-acetylcysteine (NAC) has been a well-known antidote for acetaminophen toxicity for the last 30 years [1]. Easily available over the counter, it has antioxidant and anti-inflammatory properties and an established tolerance and safety profile [2-4]. Because of its neuroprotective effects, its clinical use has recently been expanded to include the treatment of different psychiatric and non-psychiatric disorders [1]. The NAC is a synthetic N-acetyl derivative of the endogenous amino acid L-cysteine, which is a precursor of the antioxidant enzyme glutathione. The glutathione (γ-L-glutamyl-L-cysteinyl-glycine) or glutathione (GSH) acts as the body’s defense mechanism against the oxidant stress in several metabolic and pathological reactions [4]. It exerts its antioxidant effects by regulating oxidative metabolism and glutamate transmission and plays a rate-limiting role in the synthesis of glutathione, a naturally occurring antioxidant [4-5].

Limited evidence has implicated reactive oxygen species as a culprit in the neuronal dysfunction responsible for several psychiatric and neurodevelopmental conditions [4]. In view of the destructive neuronal effects of reactive oxygen species, the recent research has explored the clinical efficacy of NAC in different neuropsychiatric conditions. The NAC has been found to permeate the blood-brain barrier and has good bioavailability [4]. After intravenous administration, the mean elimination half-life of NAC is 5.6 hours in adults and 11 hours in neonates [6]. It exerts its neuronal influence by converting L-cysteine to cysteine, facilitating its uptake by glial cells and consequently allowing the glutamate release, which in turn stimulates inhibitory glutamate receptors [4]. Due to these metabotropic glutamate receptors, there is a reduction in the vesicular release of glutamate, resulting in the decrease in glutamatergic neurotransmission [5]. This mechanism of action of NAC is particularly important because of its effect on glutaminergic neurons in the nucleus accumbens, which is involved in the modulation of the reward and reinforcement center implicated in the addictive behaviors [2]. 

Several studies have associated the NAC with a reduction in the severity of the symptoms of schizophrenia, bipolar disorders, Alzheimer’s, autism and substance abuse (cannabis and cocaine) [6]. In this review, we briefly present recent progress in the use of NAC for different psychiatric disorders in the pediatric population. We also aim to highlight the characteristics of the patient populations, the outcome measures, drug regimens and adverse events reported in the trials reviewed here. Table 1 summarizes the characteristics of the studies we included, the doses of NAC, the results, and the limitations.

**Table 1:** Summary of the sample sizes, doses of N-acetylcysteine, results, and the limitation of randomized controlled trials. ASD: Autism spectrum disorder, NAC: N-acetylcysteine, MCQ: Marijuanna Craving Questionnaire, SSRI: selective serotonin reuptake inhibitors.

Study	Condition	Sample size	Dose of NAC (mg/day)	Duration	Results of study	Limitations	Adjunct medications
Hardan, et al. 2012	ASD	33	1st week- 900 mg/ day 2nd week - 1800 mg/day 3rd week- 2700 mg three times a day	12 weeks	Improvement in irritability with NAC. Decreased episodes of repetitive/stereotype behavior	Small sample size Concomitant use of psychotropic medications and behavioral interventions	Use of medications, most commonly 2nd generation antipsychotics and SSRIs
Ghanizadeh & Moghimi-Sarani 2013	ASD	40	1200mg	8 weeks	Improvement in irritability with NAC & Risperidone	Small Sample size Short duration Low dose of NAC	Medications with glutamatergic effects was not allowed Stable doses of other medications
Nikoo, et al. 2015	ASD	40	Dose of risperidone was from 1 and 2.0 mg/d, and the dose of NAC was 600 to 900 mg/d	10 weeks	The significant reduction in irritability with NAC & Risperidone. Lack of improvement in lethargy/social withdrawal subscale. No improvement in stereotypic behaviors. Reduction in hyperactivity and non-compliance. No improvement in inappropriate speech	Short duration of study Small sample size	Taking concomitant medications with glutamatergic effects were not allowed
Wink, et al. 2016	ASD	31	Dose of NAC was 300- 600 mg/ daily target dose of 60 mg/kg/day, reaching to 4200mg/day	12 weeks	No improvement in social impairment. No difference in GSH/GSSG ratio, strand break, oxidative damage of deoxyribonucleic acid (DNA), and blood homocysteine between both groups	High attrition rates Small sample size. Higher IQ in majority of participants	Stimulants, alpha-2 agonist, antipsychotics, sleep aids, antidepressants, anti-epileptic medications
Dean, et al. 2016	ASD	102	500 mg	6 months	No significant improvement were found on the primary outcomes of communication, social interaction, repetitive behaviors and parent/clinical global impression	Low dose of NAC. High attrition rates	
Bloch, et al. 2013	Trichotillomania	39	600 mg at dinner for one week, then 600 mg twice a day for one week, then 600 mg in the morning and at dinner for one week, and then remained on a dose of 1200 mg twice a day for the remainder of the 12-week study	12 weeks	No significant changes in hair-pulling severity were reported over the follow-up period	Strong possibility of Type I and II error without appropriate statistical correction	SSRIs, antipsychotics, psychostimulants, and atomoxetine
Ghanizadeh, et al. 2013	Nail biting	42	800mg/day	2 months	Lack of improvement in nail-biting	Higher rates of loss to follow-up. Psychiatric comorbidity lower doses of NAC shorter duration of exposure	Were allowed to continue medications. Detail was not mentioned
Bloch, et al. 2016	Tourette syndrome	39	2400 mg	12 weeks	No significant difference between NAC and placebo in reducing tics	Inadequate assessment of compliance. Difference in the severity of anxiety at baseline between placebo and intervention group	Antidepressants, stimulants, antipsychotics and alpha 2 agonists
Grey, et al. 2012	Cannabis use	116	2400 mg	8 weeks	Weekly negative urinary cannabinoid tests shows the beneficial effect of NAC. Improvement in self-reported days of cannabis use and craving favored NAC, though without reaching statistical significance	Short duration. Single site study	Not mentioned
Roten, et al. 2013	Cannabis-related cravings	89	2400 mg	8 weeks	A significant improvement in marijuana craving questionnaire scores over the course of the treatment, there was no significant differential change between the NAC and placebo groups	MCQ was validated in adult non-treatment seekers but it was used to measure craving in our study in adolescent treatment seekers	Not mentioned
Roten, et al. 2015	Neurocognitive performance in cannabis users	78	2400 mg	8 weeks	Significant improvement in cognition like verbal memory, composite memory and psychomotor performance with NAC	Less power to detect differences. The absence of data about cognitive performance prior to using marijuana. Absence of non-marijuana using controls	Not mentioned
McClure, et al. 2014	Smoking cessation in cannabis users	116	2400 mg	8 weeks	No change in cigarettes per day for either NAC or placebo groups	High attrition rates Smoking cessation was not primary outcome	Not mentioned

## Review

Autism spectrum disorder

Oxidative stress due to glutathione deficiency has been hypothesized to be a potential causal factor in the pathogenesis of autism spectrum disorder (ASD) [5]. The glutathione has also been linked to the frequent gastrointestinal and immunological dysfunctions in the people with autistic disorder [5]. Therefore, the NAC can serve as a potential treatment option because of its important rate-limiting role in glutathione metabolism. 

A search in PubMed with the search strategy (“N-acetylcysteine” or “NAC” and “autism”) yielded five randomized controlled trials (RCTs). In a double-blind randomized controlled trial by Hardan, et al. (2012), the efficacy of the oral NAC was assessed in physically healthy males and females aged three to 12 years with ASD, between March 2009 and September 2010 [5]. The primary outcome measures were the Aberrant Behavior Checklist (ABC) score to assess irritability and the Dosage Record Treatment Emergent Symptom (DOTES) scale score to record the severity of the side effects [5]. The participants were randomized to the NAC treatment (n=14) or a placebo group (n=15) in a 12-week double-blind RCT. The initial dose of NAC was 900 mg per day for the first four weeks, which was increased to 900 mg twice daily for the next four weeks, and 900 mg three times per day for the following four weeks. The clinical evaluation was done at the baseline and at weeks four, eight, and 12. This study reported a significant improvement in irritability in the NAC group, manifested as a lower ABC irritability subscale score and a decrease in the number of repetitive/stereotyped behaviors (Cohen’s d=0.96) [5]. Although this RCT was statistically underpowered, the authors reported low bias related to randomization, allocation concealment, attrition and blinding of the participants and personnel toward the outcome assessment. There was no statistically significant difference between the placebo and intervention groups in the frequency of the side effects.

Ghanizadeh & Moghimi-Sarani (2013) studied the efficacy of the NAC as an adjuvant in children and adolescents with autism who were treated with risperidone to decrease irritability in an eight-week double-blind RCT. The participants of both genders aged between 3.5 to 16 years were recruited from the outpatient psychiatry clinics if they had no concomitant medication prior to the enrollment. The intervention group received a combination of risperidone and NAC (1200 mg/day) in two divided doses, while the control group received a placebo with risperidone. The initial dose of risperidone was 0.5 mg/day and was gradually titrated to 2 mg/day for the participants weighing less than 30 kg and 3 mg daily for those weighing > 30 kg. The change in the ABC irritability subscale score was the primary outcome measure. This study suggested that the use of the NAC as an oral supplement and adjuvant to the current treatment for irritability was superior to the combination of risperidone and placebo (effect size = 1.4). Mild adverse effects were constipation, increased appetite, fatigue, nervousness and daytime sleepiness [6]. There was low bias related to randomization, allocation concealment and blinding of the patients, parents, and assessors, but this study too was underpowered and had attrition bias.

The effectiveness of the NAC as an adjunct to risperidone was assessed in another randomized, double-blind, controlled trial [7]. The irritability was assessed with the ABC irritability subscale in 40 children (after attrition) who were randomized to receive risperidone plus NAC or risperidone plus placebo. The secondary outcome measures included the social withdrawal, stereotypic behaviors, hyperactivity and inappropriate speech. The patients in both groups received risperidone at an initial dose of 0.5 mg with stepwise titration of 0.5 mg/week for the first three weeks. The maximum dose of risperidone was 1 mg/day for children weighing less than 20 kg and 2 mg/day for those weighing ≥ 20 kg. The intervention group received 600 to 900 mg/day of NAC in three divided doses of 200 mg/dose for children weighing < 20 kg and 300 mg/day for those weighing ≥ 20 kg. The N-acetylcysteine was effective in improving irritability compared to the placebo in this study (Cohen’s d=1.06). In addition, the hyperactivity/noncompliance improved significantly in the intervention group compared to the placebo group. The side-effect profile was mild and transient, with no substantial differences between the intervention and placebo groups [7]. This study had a low risk of bias related to randomization, allocation concealment and blinding of the participants and the study personnel. However, significant bias was likely because of low statistical power and attrition bias.

Wink, et al. (2016) designed a double-blind, placebo-controlled RCT to assess the effectiveness of the NAC in improving social communication in the patients with autism. The participants were children between the ages of four to 12 years who weighed ≥ 15 kg, and who had a confirmed diagnosis of the autism, Asperger’s disorder or pervasive developmental disorder not otherwise specified. All were judged to be moderately ill based on the Clinical Global Impression severity (CGI-S) subscale. The participants were allowed to take their concomitant medications except for glutaminergic pathway modulators [4]. The patients were prescribed a formulation of the NAC containing 300–600 mg in divided doses, with a daily target dose of 60 mg/kg and a maximum dose of 4200 mg/day. The participants who weighed 15–30 kg were started on 300 mg/day, and those who weighed more than 30 kg were started on 600 mg/day. The initial dose was continued for the first three weeks and then gradually increased and continued for the nine-week study period. The primary outcome measure was social impairment according to the CGI improvement (CGI-I) subscale. This study found no significant improvement in the social impairment in children with autism who were treated with the NAC compared to the placebo, although a marginally significant improvement was seen at week eight with a P value of 0.07 [4]. This study also investigated the impact of NAC on oxidative stress markers in peripheral and venous blood samples collected at baseline and week 12. The glutathione (Cohen’s d=0.64) and glutathione disulfide (GSSG) (Cohen’s d=0.88) improved more in the intervention group than the placebo group. However, there was no difference between groups in GSH/GSSG ratio, strand breaks, DNA oxidative damage or blood homocysteine concentration [4]. A low risk was reported in connection with randomization, allocation concealment and or blinding of the patients, parents, and personnel, but the study was biased due to attrition and beta error.

Another placebo-controlled RCT assessed the efficacy of the NAC for core symptoms of ASD as the primary outcome measure. The outcomes were assessed with the Social Responsiveness Scale, Children’s Communication Checklist (second edition), and the Repetitive Behavior Scale-Revised. A total of 102 children was allocated to the NAC or placebo group, and a dose of 500 mg/day for six months were used in the NAC group. This trial failed to show a statistically significant benefit of the NAC in the management of core symptoms of the ASD. The side-effect profile was similar in both the intervention and placebo groups [8]. The study had low statistical power and a high rate of attrition, but there was the low risk of bias in randomization, allocation concealment, and blinding of the patients and research personnel.

Trichotillomania

Trichotillomania is an impulse control disorder characterized by the chronic hair-pulling, distress and functional impairment [9]. The hair-pulling, which can occur in any area of the body where hair grows, most commonly involves the scalp followed by the eyelashes and eyebrows [10]. There is limited evidence for the effectiveness of the NAC treatment in pediatric and adult populations, and the results published to date have been mixed [11-14]. However, one study found that the NAC decreased the extracellular concentration of the glutamate in the nucleus accumbens, reducing the symptoms of compulsive behaviors such as trichotillomania [15].

A PubMed search with keywords (“N-acetyl cysteine” or “NAC” and “trichotillomania”) yielded 14 articles including one RCT that met our inclusion criteria. A double-blind, placebo-controlled trial by Bloch and colleagues enrolled 39 children and young adults and found no significant difference in the reduction of hair-pulling between the NAC and placebo groups [13]. In view of the mixed results, larger RCTs are needed to assess the effectiveness of the NAC for treating trichotillomania.

Pathological onychophagia

Onychophagia, a behavioral disorder in the children and young adults, is characterized by a compulsion leading to habitual nail-biting. By the age of 18 years, the frequency of this behavior decreases, but it may persist in some adults. It is an under-recognized problem with a three-month prevalence of 22.3% in the males and females [16]. The pharmacological options to treat nail-biting or onychophagia are limited [16]. Using the keywords (“N-acetylcysteine” or “NAC” and “onychophagia”), we were able to identify one suitable RCT out of six search entries in the PubMed.

There is limited evidence for the efficacy of the NAC in onychophagia. A study by Ghanizadeh, et al. (2013) explored the efficacy of the NAC in the treatment of nail-biting in a two-month double-blind, placebo-controlled trial [16]. This study recruited 25 children and adolescents aged six to 18 years, with a history of chronic nail-biting that caused emotional distress and physical damage but without serious medical illness. Concomitant medications were allowed during the trial, and participants were informed about the primary outcome and requested not to cut their nails. In the NAC group, nine children received concurrent medication, including citalopram, fluoxetine, nortriptyline, methylphenidate, risperidone, and atomoxetine. The N-acetylcysteine was given at the dose of 800 mg/day with an initial dose of 200 mg/day. The outcome measure was the length of all nails in millimeters, measured twice in each session. A headache and aggressive behavior were the most common adverse effects in the NAC group. This study suggested that nail length increased more gradually in the NAC treatment group compared to the placebo group during the first month, but no significant difference was found in a follow-up assessment after two months. The N-acetylcysteine was thus found to be effective in improving impulsiveness in nail-biting for a short period [16]. The study had low power and high risk associated with attrition, but was double-blinded and randomized, and had low risk associated with allocation concealment. It also had a few limitations: a) high levels of psychiatric comorbidity, (b) low doses of NAC administered, and (c) short duration of the treatment. Due to its very small sample size, no cause-effect relationship related to the adverse effects of medication could be inferred from this study.

Pediatric Tourette syndrome

Tourette syndrome is a chronic disorder that starts in childhood and is characterized by motor and vocal tics of variable frequency, persisting for more than one year [9]. It is often associated with behavioral disorders, particularly obsessive-compulsive disorder (OCD) and attention deficit hyperactivity disorder (ADHD). The PubMed searches with the keywords (“N-acetylcysteine” or “NAC” and “Tourette”) yielded one suitable RCT for inclusion out of eight entries. 

Considering the potential evidence in the spectrum of OCD, Bloch, and colleagues (2016) studied the effectiveness of the NAC for the management of the pediatric Tourette syndrome in a double-blind, placebo-controlled trial [3]. In their study, 31 children and adolescents aged eight to 17 years were randomly allocated to receive NAC or a placebo for 12 weeks [3]. A significant proportion of the participants had a comorbid disorder such as OCD and ADHD and were receiving concurrent treatments such as psychological therapy, antidepressants, stimulants, antipsychotics and alpha-2 agonists. The primary outcome measures were the Yale Global Tic Severity Scale (YGTSS) score and the Total tic score. There was no significant difference between the NAC and placebo groups in the reduction of tic severity [3]. Like previously reported studies, this study was underpowered; however, other methodological characteristics were of adequate quality. A headache was the only adverse effect, reported by one participant in the NAC group. Inadequate assessment of compliance and differences between the placebo and intervention groups in the severity of anxiety at baseline were major limitations in this study.

Cannabis use disorder

Substance use disorder is defined as the recurrent use of alcohol and/or drugs that can cause functional impairment such as health problems, disability, and failure to meet major responsibilities at work, school or home. According to the Diagnostic and Statistical Manual of Mental Disorders (DSM)-5, a diagnosis of substance use disorder is based on evidence of impaired control, social impairment, risky use and pharmacological criteria [9].

A PubMed search with the keywords (“N-acetyl cysteine” and “cannabis”) yielded 13 articles, including four that met our inclusion criteria. The efficacy of NAC as a pro-drug for cannabis use disorder and nicotine use disorder was studied in one clinical trial [2]. In this large double-blind randomized trial, Gray, et al. administered 2400 mg/day of NAC or placebo for eight weeks with 116 participants between 15 and 21 years old who were addicted to marijuana [2]. Both groups also received a contingency management intervention and cessation counseling. Significantly more participants in the NAC treatment group had a negative urine cannabinoid test [2]. The measures of self-reported days of cannabis use and craving favored NAC, although the differences compared to the placebo group did not reach statistical significance [2]. The significant improvements in cognitive performance were seen in the early stages of the treatment-associated abstinence [2]. 

Roten, et al. studied the role of NAC in controlling cravings in the patients who were dependent on cannabis [17]. The participants in this double-blind RCT had a history of cannabis dependence and were between 15 and 21 years old. All participants were given oral NAC at 1200 mg twice per day or a placebo for eight weeks and also received cessation counseling for less than 10 min with the management intervention [17]. The Marijuana Craving Questionnaire (MCQ) was used to assess cravings. This study did not find a decrease in marijuana craving in the NAC treatment group compared to the placebo group [17]. In part of this study published as a separate article, the role of NAC in improving cognitive performance was analyzed [18]. All participants completed a cognitive task performance test and the computer-administered computerized neurocognitive (CNS) vital signs battery test at baseline on their first appointment and in weeks four and eight during the trial were done. The authors found significant improvements in cognitive tasks such as verbal memory and psychomotor speed within weeks of marijuana cessation [18]. Further analyses of data from this trial found that the NAC treatment was associated with increased rates of abstinence in the individuals seeking treatment for cannabis use disorder, although positive correlations were also found with low impulsivity, high medication adherence, and negative urine cannabinoid test findings at baseline [18].

The co-occurrence of cannabis and tobacco use is highly prevalent and is a major public health issue. The clinical role of the NAC to treat nicotine use disorder was investigated in the patients with a history of cannabis use [19]. This study recruited 116 participants, 15 to 21 years old, who used cannabis regularly (> three days/week) and showed interest in the cannabis cessation treatment [19]. The participants were allocated to the NAC or placebo group, and the dose of NAC was 1200 mg twice per day with a matched placebo. The treatment continued for eight weeks, and follow-up appointments were held in 12 weeks. The clinical assessment was based on urine samples and the number of cigarettes per day [19]. This study suggested a decrease in urges/craving to smoke cigarettes and cannabis but did not find a reduction in the number of cigarettes smoked per day in either group.

## Conclusions

Because of its antioxidant and glutamate modulating properties, the NAC has become a focus of interest in recent studies designed to investigate the treatment options for the core symptoms of ASD and comorbid irritability. Although the evidence available to date for its therapeutic activity is marred by low statistical power, the NAC may be a potential treatment option as an adjunct to antipsychotics in efforts to improve symptoms of irritability. Recent RCTs has yielded mixed results for different outcomes, but these studies were limited by their small sample sizes, short duration, different formulations of NAC, and different dosage regimens.

The children with ASD constitute a heterogeneous group of the patients with a range of strengths and challenges that impact the efficacy of the treatment. A few RCTs have reported improvements in repetitive or stereotyped behaviors, lethargy, and social withdrawal in addition to improvements in irritability. However, these results have generally not been replicated in other studies. Future research should be conducted with appropriate doses of NAC and longer follow-up periods to better document its potential benefits for the core treatment of ASD. The N-Acetylcysteine was ineffective for the treatment of trichotillomania, habitual nail-biting and pediatric Tourette syndrome. On the other hand, it has been tested for cannabis use disorder and smoking cessation with favorable results. However, the clinicians should carefully consider the benefits, risk factors and alternatives when recommending NAC as a treatment option. The findings of the RCTs reviewed here need to be validated by carefully planned studies with adequate sample sizes.
